# Tumour-to-tumour metastasis: male breast carcinoma metastasis arising in an extrapleural solitary fibrous tumour – a case report

**DOI:** 10.1186/s13000-014-0203-y

**Published:** 2014-11-25

**Authors:** Susanne Scheipl, Farid Moinfar, Andreas Leithner, Patrick Sadoghi, Mette Jorgensen, Beate Rinner, Bernadette Liegl

**Affiliations:** Department of Orthopaedics and Orthopaedic Surgery, Medical University of Graz, Auenbruggerplatz 5, 8036 Graz, Austria; Institute of Pathology, Medical University of Graz, Auenbruggerplatz 25, 8036 Graz, Austria; University College London Cancer Institute, 72 Huntley Street, London, WC1 6BT UK; Center for Medical Research, Cell Culture Facility, Medical University of Graz, Stiftingtalstrasse 24, 8010 Graz, Austria

**Keywords:** Tumour-to-tumour metastasis, Soft tissue tumour, Solitary fibrous tumour, Breast cancer

## Abstract

**Background:**

Tumour-to-tumour metastasis (TTM) occurs when one tumour metastasises to a separate tumour within the same individual. TTM is observed frequently in breast cancer but has not been described in male breast cancer. In addition reports describing solitary fibrous tumours (SFT) of the pleura hosting other neoplasms’ metastases are limited. We report an exceptional case of male breast cancer metastasising to an extrapleural SFT, occurring in the subcutaneous tissue of the back of a 68-year old Caucasian patient.

**Case presentation:**

A 68-year old male was diagnosed with a metastasising ductal breast cancer. He was treated by mastectomy of the right breast and axillary lymph-adenectomy. Further staging revealed an increasing subcutaneous expansion located on the patient’s back. Excision biopsy confirmed a SFT hosting a breast cancer metastasis. The patient received palliative chemotherapy but died of disease seven years after initial diagnosis.

**Conclusions:**

The abundance of blood vessels within these lesions might predispose SFTs for an involvement in TTM. This case describes the possibility of concurrent rare occurrences and reminds clinicians, as well as pathologists, to be open-minded and fastidious about their differential diagnoses, sampling and examination of histological specimens.

**Virtual Slides:**

The virtual slide(s) for this article can be found here: http://www.diagnosticpathology.diagnomx.eu/vs/13000_2014_203

## Background

Tumour-to-tumour metastases (TTM) occurs when one tumour metastasises to another tumour within the same individual [1]. TTM are rare, but well-documented findings [2,3]. Lung cancer and breast cancer are frequent donor tumours, [2,4,5] with renal cell carcinoma (RCC) being the most frequent recipient [1]. Though breast cancer is frequently involved in TTM, male breast cancer is rare, [6] and has to our knowledge not been described in the setting of TTM. There are infrequent reports describing solitary fibrous tumours (SFT) involvement, as hosts, in TTM [4,5,7]. In those cases, SFTs originated from the pleura. We first report an exceptional case of male breast cancer metastasising to an extrapleural SFT occurring in the subcutaneous tissue of the back of a 68-year old patient.

## Case presentation

A 68-year old slightly obese (BMI: 27.7 kg/m^2^) Caucasian male was diagnosed with a moderately differentiated ductal breast cancer by biopsy in March 2006. The patient was a retiree who had been employed in the national railway services throughout his working career. At the time of breast cancer diagnosis, he had also been diagnosed with and/or treated for essential hypertension, arteriosclerosis and benign prostate hyperplasia. Furthermore, he had received treatment for helicobacter-associated chronic gastritis earlier in his life. His family anamnesis of breast cancer was negative. After neoadjuvant chemotherapy, the patient was treated by mastectomy of the right breast and axillary lymph-adenectomy. Surgery was followed by adjuvant chemotherapeutic treatment and local irradiation therapy. Fourty-six months after initial diagnosis, metastases of the lung and bone were detected. Further staging demonstrated a subcutaneous expansion located at the left side of the patient’s back. The patient reported he had noticed this indolent expansion over the past fifteen years. During the last twelve months, there had been minor progression. In order to exclude soft-tissue metastasis, excision biopsy was performed at an external hospital 47 months after the initial breast cancer diagnosis.

**Figure 1:**
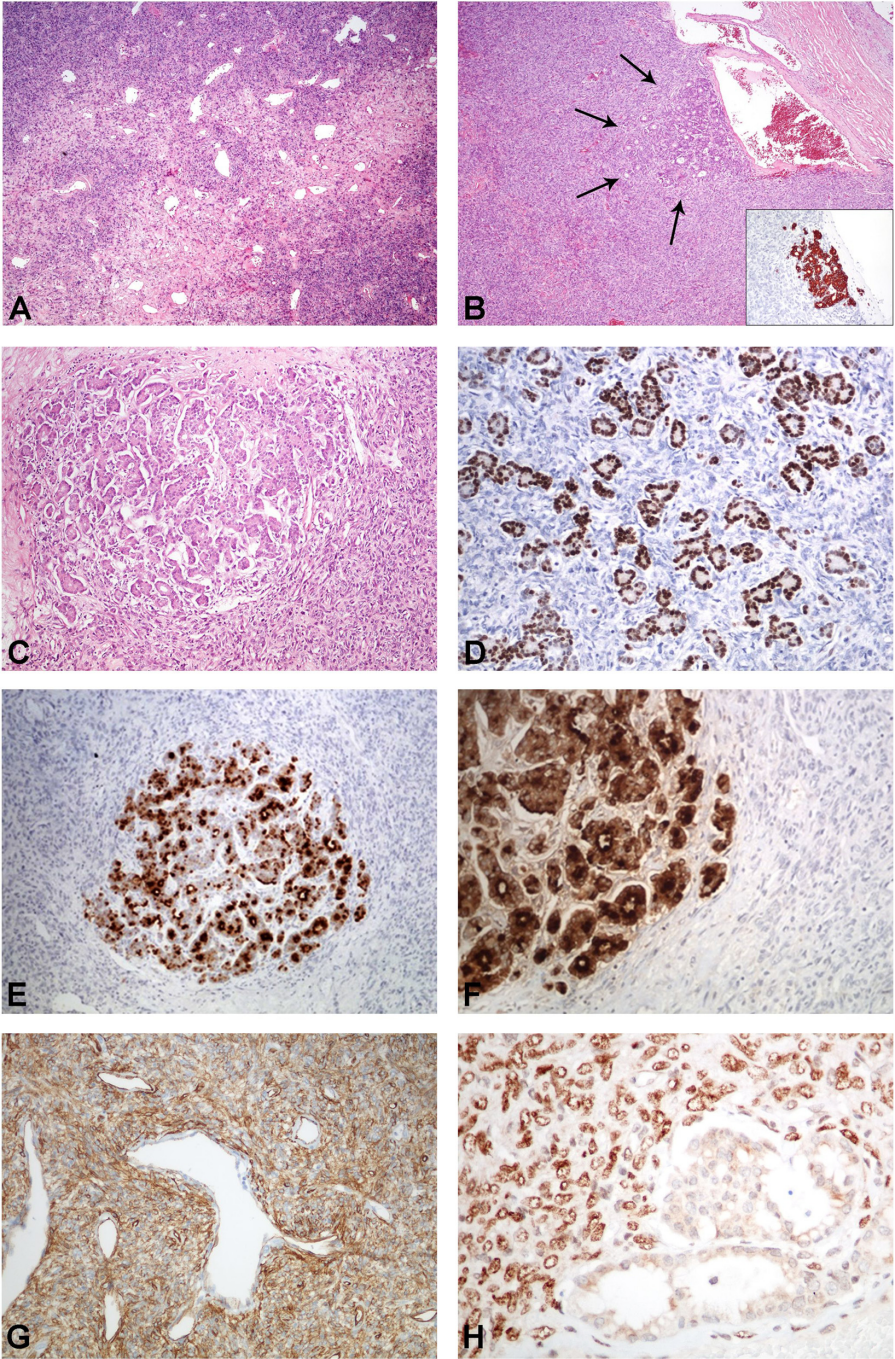
**“Extrapleural SFT hosting a male breast cancer metastasis”. A)** Classic morphology of a SFT. **B)** Ductal structures within a SFT representing the metastasis of a male ductal adenocarcinoma of the breast. The ductal adenocarcinoma shows strong CK7 expression (insert). **C)** Higher power view of the metastasis within the SFT. **D**-**F)** The ductal adenocarcinoma is positive for estrogen **(D)**, BRST2 **(E)**, and mammoglobin **(F)**. **G)** The SFT component shows strong CD34 expression. **H)** The SFT expresses STAT6.

*Macroscopic examination* revealed a solid, grayish lesion with a maximum diameter of 8 cm. Histological examination showed a mesenchymal tumour composed of a mix of round-oval to spindle shaped tumour cells with indistinct cell borders (Figure 1A). The tumour cells were set within a collagenous stroma, and arranged in a random pattern. Mast cells were present. A prominent “haemangiopericytoma-like” vascular architecture was seen. The mesenchymal tumor component showed focal mild nuclear atypia. Necrotic areas were not present and mitoses were scarce (3/10 HPF). Within the tumor, nests of epithelial cells and ductal structures were seen (Figure 1B and C). The epithelial tumor component demonstrated only mild to moderate nuclear atypia.

The mesenchymal spindle cell component showed strong *immunohistochemical* positivity for antibodies (Ab) against CD34 (Figure 1G) and STAT6 (Figure 1H) and was negative for various cytokeratins (CKs), SMA, Desmin, S100 and EMA. The ductal epithelioid structures showed strong positivity for Ab against various CKs, particularly CK7, (Figure 1B, insert), EMA, BRST2 (Figure 1E), Mammaglobin (Figure 1F), estrogen (Figure 1D), but not progesterone. Also, HER-2/neu overexpression was detected. The diagnosis of a SFT hosting a metastasis of a male breast cancer was made (Figure 1B).

*Molecular analysis* of the breast cancer tissue for BRCA1 and BRCA2 mutations revealed a wild-type status for these two genes. Five sequence variants were detected (BRCA1 S1634G, K1183R, E1038G, P871L and BRCA2 V2466A), which were described as naturally occurring polymorphisms in publicly available databases (1000 Genomes and dbSNP).

The patient received palliative chemotherapy but died of disease seven years after initial breast cancer diagnosis and 36 months after TTM resection, respectively.

### Discussion

Metastasis of one tumour to another tumour within the same individual is a rare, but well-documented finding [1,3]. TTM have first been described by Berent in 1902 [2,4]. Rabson (1954), [8] Dobbing (1958), [9] Gore and Barr (1958), [10] and Campbell (1968) were among the first to review these rare occurences and to define criteria for diagnosis [1,4]. Not only these authors, but many others after them, have reported RCC as most frequent recipient [1,4,7]. This is thought to be due particularly to the excellent vascularity of this tumour combined with the high blood supply of the organ of origin, together making it more likely for circulating emboli of other tumours to be caught in RCCs [1,4,5,9]. Among the donor neoplasms, lung cancer has been described as the most common primary, but also breast, prostate, and thyroid carcinomas have frequently been reported to be involved in TTM [5,7].

Although breast cancer is known to be a frequent donor of TTM, [2,5] male breast cancer is an exceptional finding with a reported incidence of less than 1% of male cancers [6]. Due to its rarity, diagnosis is often delayed and prognosis is poor. Thus, metastatic spread is often observed at the time of diagnosis [6]. Yet, to the best of our knowledge, there have been no reports referring to male breast cancer metastasising to other tumours.

Apart from epithelial tumours, sarcomas and mesenchymal tumours have been described as donors and/or recipients in TTM [2,4,7,11,12].

SFTs have been mentioned in the setting of TTM previously: once hosting the metastasis of an urothelial carcinoma of the bladder, [7] second being recipient to a metastasis of RCC, [4] and third hosting a breast cancer metastasis in a female [5]. However, all of these cases refer to pleural SFTs. Though initially described in the pleura, SFTs can occur in almost any site [13]. Their biological behavior varies from benign to malignant, and is not predictable in the individual case [13]. The abundance of blood vessels within these lesions might predispose SFTs to filter microemboli from other tumours, thus increasing the likelihood of their involvement in TTM.

## Conclusion

In conclusion, this case describes the possibility of concurrent rare occurrences and reminds clinicians, as well as pathologists, to be open-minded and fastidious about their differential diagnoses, sampling and examination of histological specimens.

## Consent

Written informed consent was obtained from the patient’s widow for publication of this Case report and any accompanying images. A copy of the written consent is available for review by the Editor-in-Chief of this journal.
